# Dispositional Optimism in Christian Populations Compared to other Religious Groups: a Scoping Review

**DOI:** 10.1192/j.eurpsy.2023.2108

**Published:** 2023-07-19

**Authors:** W. W. L. Cheung, C. Liu, V. W. L. Tsang

**Affiliations:** 1 University of British Columbia, Vancouver, Canada; 2 Harvard T.H. Chan School of Public Health, Boston, United States

## Abstract

**Introduction:**

There are various ways people cope with life events. One can expect generalized positive or negative outcomes across various life domains, called dispositional optimism. This can be explained by attribution theory: how people explain past events, their causations, and outcomes. Understanding attribution styles is important to help people reframe current circumstances and improve mental wellbeing. Our hypothesis is that people of different religious groups may exhibit various levels of optimism and pessimism based on their values, teachings, and practices. Previous research has found that people of Christian faith, or those with a religious faith in general, look to their religion as a way of coping during life adversities. Certain religious practices such as prayers and Church gatherings have been found to improve mental health through increasing dispositional optimism. While the relationship between religiosity and mental health has been previously examined in different religious populations, there are few studies that focused on comparing this relationship across religions.

**Objectives:**

The objective of this scoping review is to understand the link between religiosity and mental health, focusing primarily on how people of the Christian religion demonstrate dispositional optimism or pessimism when coping with adverse life events, compared to other religious groups or atheists.

**Methods:**

This scoping review included original peer reviewed study articles that studied mental health in terms of dispositional optimism or pessimism in people of Christian religion compared to other religious groups. This review used online databases, Ovid MEDLINE and PsycInfo, and used extraction tables to analyze the results of past research.

**Results:**

The results of this scoping review revealed that people of Christian religion, especially those high in religiosity, use their religion as a method of coping. This population also showed higher dispositional optimism compared to atheists or those that believe in other religions. However, when compared to other religions such as Buddhism and Muslim, Christian populations showed lower dispositional optimism.

**Conclusions:**

It is evident that religious involvement is linked to aspects of mental health, but comparing the effects of different religions is still a topic of exploration that can be investigated further to allow deeper understanding of their similarities and differences, as well as the mechanisms by which religion can affect mental health. In this review, a gap in the body of knowledge regarding the relationship between religion and pessimism was revealed. Future research directions could include examining whether dispositional pessimism varies across religious groups, as it does not necessarily have a perfectly inverse relationship with optimism.

**Disclosure of Interest:**

None Declared

